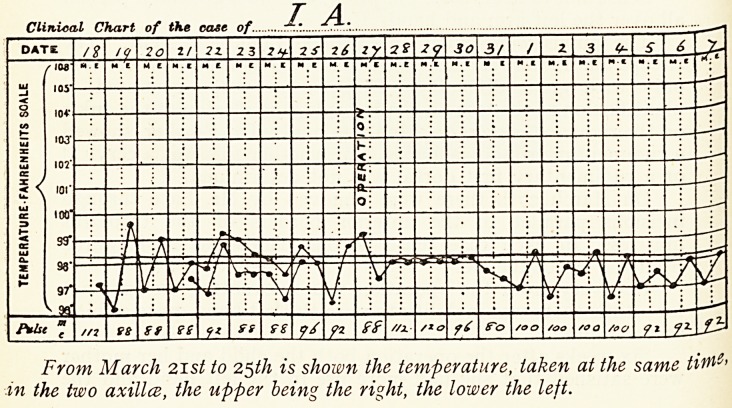# A Case of Cervical Rib

**Published:** 1913-09

**Authors:** Rupert Waterhouse

**Affiliations:** Physician to the Royal Mineral Water Hospital; Pathologist and Assistant Physician to the Royal United Hospital, Bath.


### A CASE OF CERVICAL RIB.

BY

RUPERT WATERHOUSE, M.D., M.R.C.P.,

*Physician to the Royal Mineral Water Hospital; Pathologist and Assistant Physician to the Royal United Hospital, Bath.*

---

I. A., aged 12, came to the Royal United Hospital on March 10th, 1913, complaining that seven months previously she began to suffer severe pain of a shooting character in the region of the left shoulder. After a week or two the pain passed down into the left arm and fingers, where it remained until recently, though gradually diminishing. Shortly after the onset of pain it was noticed that her left hand was weak, and this has gradually got weaker and smaller since. The left hand and arm always feel cold, and sometimes the hand would go "dead," but no other unusual sensations, such as "pins and needles," have been noticed. She had not been laid up in bed, nor was there any history of injury. Shortly before admission pain was complained of in the right shoulder and down the front of the thighs.

Since the age of five she had been subject to diarrhœa, seldom getting through a summer without two or three attacks,

*Photograph of left hand, showing wasting of thenar eminence and of the abductor muscles of the thumb.*

*Skiagram of cervical region taken by Dr. Bowker, showing a rudimentary rib on either side.*

and sometimes being under treatment two or three months at a time. Last January she had an attack of "catarrhal jaundice," followed by very intractable diarrhœa. There have been no other illnesses.

The child was bright and intelligent for her age, well formed, and except for slight pallor appeared well nourished. The left hand showed absence of the thenar eminence, with marked flattening of the hypothenar and some hollowness between the metacarpal bones. The fingers of the left hand could not be completely extended, but all other movements of the hand and arm could be performed, though less strongly than on the right. There was paresis of the serratus magnus on both sides, so that on horizontally extending the arms in front of the body the vertebral borders of both scapulæ stood out from the thorax.

The dynamometer showed the grip of the left hand to be 15, that of the right 32. The girth of the left arm at its middle was $\frac{3}{4}$ in. less than that of the right (R.=$7\frac{1}{2}$, L.=$6\frac{3}{4}$), and that of the forearm 2 in. below the olecranon $\frac{7}{8}$ in. less on the left (R.=$7\frac{5}{8}$, L.=$6\frac{3}{4}$).

The left hand and arm were distinctly colder to touch than the right, the surface temperature of the outside of the arm being 89.5 on the left and 93 deg. on the right, whilst, as will be seen from the accompanying chart, the temperature in the left axilla was constantly lower than that in the right.

The muscles of the arm and forearm reacted to faradism, but a stronger current was required on the left than on the right. None of the muscles of the left hand reacted, except the fourth dorsal interosseous, but they responded to galvanism, though a stronger current was required on the left than on the right.

Sensation to heat, cold, pin-prick, and wool appeared perfect all over the left arm and hand.

The pupils were equal and reacted briskly to light. The triceps and radial reflexes were not obtained, and the knee-jerks only with great difficulty. The plantar reflex consisted of plantiflexion of the toes on the left side, dorsiflexion of the ankle on the right. A skiagram, kindly taken by Dr. Bowker, showed a rudimentary rib on each side, but whether this was a cervical or a rudimentary first dorsal was not determined.

On March 27th these were removed by Mr. Lace through incisions parallel to the anterior borders of the trapezius muscles. In each case tense fibrous bands passed forwards from the tip of the rib.

Recovery from the operation was uneventful, except that for a time there was inability to raise the left hand quite as high as the right.

Six weeks after the operation both the child and her mother were satisfied that she had more use in the left hand and the feeling of coldness had passed off, nor was the limb cooler to the touch than its fellow. Indeed, the temperature in the left axilla was distinctly higher than in the right (R.=98.2, L.=98.9). The power of the grip had not increased (R.=30, L.=15). The girth of the left arm at its middle was $\frac{1}{2}$ in. less than that of the right (R.=$7\frac{3}{4}$, L.=$7\frac{1}{4}$), and that of the forearm 2 in. below the olecranon $\frac{1}{4}$ in. less on the left (R.=$7\frac{7}{8}$, L.=$7\frac{5}{8}$).

There are several points of interest in this case. In the first place, paralysis of the posterior thoracic nerve, present in this case on both sides, appears to receive no mention in cases of cervical rib that have been recorded.

The differences in temperature of the two limbs was probably the result of pressure on the sympathetic fibres in the cords of the brachial plexus, causing a widespread vaso-constriction. This extensive vaso-constriction, apart from actual muscular wasting, probably plays no small share in causing diminution in size of the more affected limb.

With regard to the hand muscles, the wasting was altogether out of proportion to the loss of power, causing a resemblance to the state of affairs met with in progressive muscular atrophy.

The slight irregular pyrexia which preceded the operation is interesting, as seeming to indicate that actual inflammatory changes were taking place in the nerves of the brachial plexus as the result of pressure.

*Clinical Chart of the case of* I. A.

*From March 21st to 25th is shown the temperature, taken at the same time, in the two axillæ, the upper being the right, the lower the left.*

## Figures and Tables

**Figure f1:**
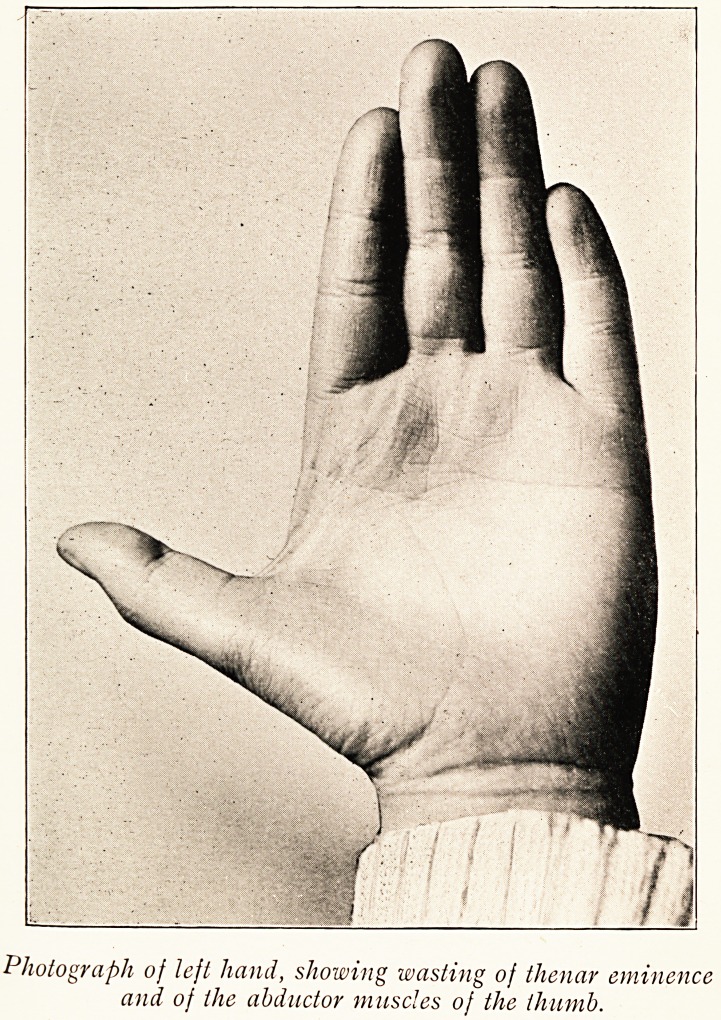


**Figure f2:**
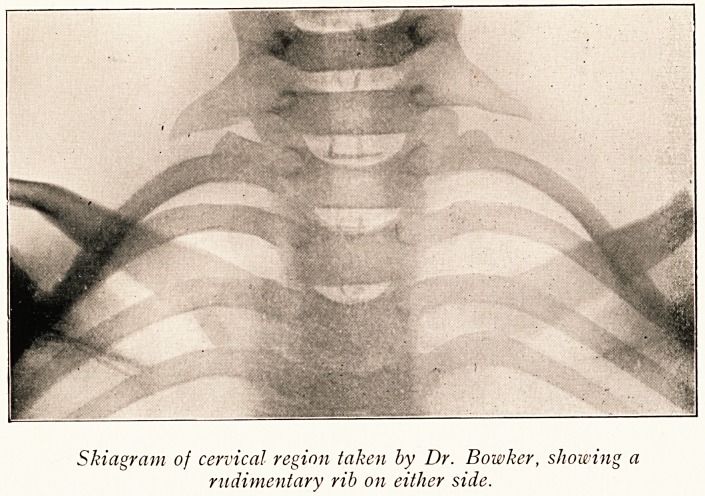


**Figure f3:**